# Unmet need for assistance with activities of daily life among older adults in Brazil

**DOI:** 10.11606/S1518-8787.2018052000463

**Published:** 2018-07-13

**Authors:** Tania Bof de Andrade, Fabiola Bof de Andrade

**Affiliations:** I Faculdade de Ciências Médicas de Minas Gerais. Programa de Pós-Graduação em Geriatria. Belo Horizonte, MG, Brasil; II Fundação Oswaldo Cruz. Instituto de Pesquisa René Rachou. Belo Horizonte, MG, Brasil

**Keywords:** Aged, Disabled Persons, Activities of Daily Living, Health Services Needs and Demand, Old Age Assistance, Socioeconomic Factors, Health Inequalities

## Abstract

**OBJECTIVE:**

To estimate the proportion of unmet need for personal assistance for basic and instrumental activities of daily life and to evaluate socioeconomic inequalities related to the unmet need among community-dwelling Brazilian older adults.

**METHODS:**

This was a cross-sectional study with data from the last National Health Survey in Brazil. Unmet need was considered as the presence of at least one unmet need for basic or instrumental activities of daily life among individuals reporting the need for assistance. Logistic regression models were used to assess the correlates and probabilities of unmet need.

**RESULTS:**

The proportion of unmet need was 18% and 7.1% for basic and instrumental activities of daily life, respectively. Unmet need was significantly related to living arrangements and socioeconomic status. Individuals in the first quintile of wealth status had about 50% higher probability of having an unmet need. A family member was the most prevalent type of caregiver.

**CONCLUSIONS:**

Long-term care policy is needed to reduce the proportion of unmet need, especially among socioeconomically disadvantaged groups. Future studies should address the availability, training, and remuneration of caregivers, as those are an indispensable labor force in an aging society.

## INTRODUCTION

The increase in life expectancy has raised the number of older individuals living in the community with disabilities and with a higher risk of dependence, who need frequent human help or care beyond the kind habitually required by a healthy adult^1^. As a result, the aging population is increasing the attention to the challenges and demands for long-term care^2^, especially in low- and middle-income countries, where most older individuals will live^2^.

Long-term care are the activities undertaken by informal (family, friend, or neighbors) or formal (health and social professionals or paraprofessionals) caregivers to ensure that people with or at risk of an ongoing loss of intrinsic capacity can maintain a level of functional ability and quality of life^2^ ^,^ ^3^. Accordingly, unmet need for personal assistance for activities of daily life is related to increased risk of mortality^4^ and morbidity (e.g. falls, weight loss)^5^, hospitalization and re-hospitalization^6–8^, and psychological distress^9^.

Unmet need for basic activities of daily life (BADL) was found to range from 14% in the United States^8^, to 61% in China^4^. Regarding instrumental activities of daily life (IADL) unmet need ranged from 18% in the United States^5^, to 40%, among women in Montreal^9^. Moreover, the main source of support and care worldwide is the family^2^ ^,^ ^10^, which is becoming a critical aspect for the success of aging in place, given the changes in family structures and the lack of public community-based long-term systems^3^. In Brazil, less than 1% of the older adults are in long-term care institutions. Although healthcare is delivered free of charge through its public health system, there is no national policy for home-based long-term care^11^. Understanding the correlates of unmet care is not only essential for planning long-term policy^12^ ^,^ ^13^, but also for avoiding poor health outcomes.

Regarding factors related to unmet need, associations were found in relation to sociodemographic factors^14^, level of disability^12^ ^,^ ^15^, and living arrangements^12^ ^,^ ^1^ ^4^ ^,^ ^15^, which is the most consistent relationship. As few studies have investigated the unmet need for activities of daily life, its correlates remain inconclusive. Moreover, most of the studies were conducted in developed countries.

In Brazil, there is no national estimate of the proportion of unmet need regarding BADL and IADL. A previous study investigated the prevalence of formal and informal care for overall activities of daily life. In addition, the study investigated the prevalence of lack of any help compared with help provided by formal and informal caregivers^16^. As each group of activities generates different care demands, there is a need for information regarding both activities for future policy planning. BADL often implies the need for direct assistance from others, which is expensive and complex. On the other side, as IADL are related to community life, they are less related to a direct assistance^17^.

The present study fills these gaps and uses data from a national sample of community-dwelling older people from Brazil with the following objectives: 1) estimate the proportion of unmet need for personal assistance for BADL and IADL; 2) evaluate socioeconomic inequalities related to unmet need.

## METHODS

This was a cross-sectional study with data from the last Brazilian National Health Survey, conducted by the Brazilian Institute of Geography and Statistics and the Ministry of Health in 2013. The sample comprised 64,348 households and was stratified into three cluster stages. The primary sampling units were census tracts, households were the second-stage units, and the adults in these households (18 years of age and over) were the third-stage units. Detailed information about the study has been published elsewhere^18^. All participants were interviewed in their own homes by interviewers specifically trained to apply a comprehensive questionnaire on sociodemographic and behavioral characteristics, lifestyle, quality of life, use of health services, and general health information.

This study included all participants aged ≥ 60 years, with disabilities in basic (n = 2,165) and instrumental activities of daily living (n = 5,762) that reported the need for help. Disability was defined as any difficult to perform one or more basic BADL (bathing, dressing, walking, using the toilet, feeding, and moving in and out of bed)^19^ or one or more IADL (managing finances, using transportation, shopping, and taking medication)^20^.

Unmet need was considered as the presence of at least one unavailable need for BADL and IADL. Individuals reporting difficulty to perform BADL or IADL were asked whether they received personal assistance to perform each activity; with three possible answers: 1) yes; 2) no, there is no need; 3) no, because there is no help available. Based on the answers, participants were categorized as: 1) met need (answered “yes”, have assistance for all activities they have difficulty performing), 2) unmet (answered “no, because there is no help available” for one or more activities with they have difficulty performing). The BADL and IADL were evaluated separately.

Independent variables assessed demographic characteristics (age [60–69 years, 70–79 years, ≥ 80 years], sex [male, female], ethnicity [white; nonwhite, i.e., black, brown, yellow, indigenous]), and living arrangement (no marital relationship and doesn’t live alone, no marital relationship and lives alone, marital relationship and doesn’t live alone); socioeconomic status (wealth [1st quintile, 2nd quintile, 3rd quintile, 4th quintile, 5th quintile]), education (0–3, 4–7, 8–11, and 12 years or more of formal education) and number of disabilities (BADL [1–2, 3–4, 5–6] and IADL [1, 2, 3–4]).

The wealth status was obtained from factor analysis of principal components^21^ using the following variables: household assets (internet [yes, no], television, refrigerator, washing machine, computer, desk phone, cell phone, microwave, motorcycle), household characteristics (maid [yes, no], coated floor [yes, no], wall of masonry or wood [yes, no], roof of tile, slab, or wood [yes, no], piped water [yes, no], sewage treatment or septic tank [yes, no], garbage collection [yes, no], crowding [number of people living in the household divided by the number of rooms used as bedroom]). The wealth status was categorized into quintiles.

Statistical analysis involved descriptive and inferential analyses, with a 5% significance level and 95% confidence interval (95%CI). The proportion of met need and unmet need were estimated among individuals with disabilities in BADL and IADL. Prevalence of the main care provider for each BADL and IADL were estimated according to the following categories: 1) family member who lives in the home and is not paid to help; 2) family member who doesn’t live in the home and is not paid to help; 3) caregiver hired to help; 4) family member who lives in the home and is paid to help; 5) non-family member who is not paid to help; 6) maid; 7) family member who doesn’t live in the home and is paid to help.

Associations between categorical variables were assessed using the Rao-Scott chi-squared test^22^. Multiple logistic regression models were fitted independently for each dependent variable, namely unmet need for BADL and IADL. The inclusion of covariates followed a hierarchical model^23^. Covariates were included in four blocks from the most distal to the most proximal to the outcome in the following order: demographic characteristics, socioeconomic status, behavior, health status. All analyses were conducted using Stata 13 (College Station, Texas, 2013), considering the complex survey design and sampling weights.

The survey was approved by the Brazilian National Commission on Ethics in Research (CONEP) of the National Health Council (CNS).

## RESULTS

The prevalence of disabilities in BADL e IADL were 16.0% and 26.6%, respectively. Among individuals with a disability in BADL, no need, met need, and unmet need were reported by 42.6%, 47.0%, and 10.3%, respectively. Regarding IADL, no need was reported by 11.6%, while 82.2% and 6.3% reported meet and unmet need, respectively. When considering only the disabled individuals with the need for personal assistance, the proportion of unmet need was 18% for BADL and 7.1% for IADL.

Table 1 shows the proportion of unmet need according to each of the activities of daily life. Unmet needs for BADL were higher than for IADL. The main care provider for both BADL and IADL situations was a family member, more specifically, a family member who lived in the home and was not paid to help (66.8% for BADL and 55.6% IADL) (Table 2).

**Table 1:** Unmet need for personal assistance according to basic (BADL) and instrumental (IADL) activities of daily life.

Activities of daily living	BADL		IADL	
	
	Difficulties (%)	Unmet (%)*	Difficulties (%)	Unmet (%)*
Basic				
Using the toilet	6.6 (6.1–7.1)	9.7 (7.2–13.0)		
Dressing	9.2 (8.6–9.8)	10.7 (8.6–13.1)		
Bathing	7.5 (7.0–8.1)	10.9 (8.2–14.3)		
Feeding	5.0 (4.6–5.4)	11.7 (8.8–15.4)		
Moving in and out of bed	8.8 (8.2–9.4)	15.0 (12.2–18.4)		
Walking	8.8 (8.2–9.4)	15.9 (12.7–9.7)		
Total	16.0 (15.2–16.8)	18.0 (15.4–21.0)		
Instrumental				
Managing finances			12.8 (12.1–13.5)	2.3 (1.8–3.1)
Taking medication			11.4 (10.6–12.2)	3.2 (2.2–4.5)
Using transportation			22.0 (21.1–22.8)	4.5 (3.6–5.6)
Shopping			17.9 (17.1–18.7)	5.0 (4.1–6.1)
Total			26.6 (25.7–27.6)	7.1 (6.1–8.2)

* Proportion with unmet needs.

**Table 2:** Main care provider for basic (BADL) and instrumental (IADL) activities of daily life.

Main care provider	BADL	IADL
Family member who lives in the home and is not paid to help	66.8	55.6
Family member who doesn’t live in the home and is not paid to help	14.1	31.5
Caregiver hired to help	7.1	2.3
Family member who lives in the home and is paid to help	4.8	3.4
Non-family member who is not paid to help	2.8	3.6
Maid	2.6	1.4
Family member who doesn’t live in the home and is paid to help	1.8	2.2

For both BADL and IADL, most individuals were women, had 0–3 years of education, and did not have a marital relationship, but lived with others. About 43.1% had difficulties in 5–6 BADL and 46.3% had difficulties in 3–4 IADL (Table 3). According to the bivariate analysis (Table 3), unmet need for BADL was related to all independent variables, except by schooling. Unmet need for IADL was related to the same variables as BADL, but there was no association with the number of IADL.

**Table 3:** Distribution of older people with unmet needs for activities of daily living according to sociodemographic covariates.

Variable	Unmet needs			

	BADL		IADL	
	
	Total (%)	Yes (%)	Total (%)	Yes (%)
Sex				
Male	37.1	16.4	33.2	7.6^a^
Female	62.9	19.0	66.8	5.6
Age (years)				
60–69	27.7	29.3^c^	27.2	8.6^b^
70–79	29.4	21.6	35.6	6.7
≥ 80	42.9	8.3	37.1	3.8
Schooling (years)				
0–3	60.0	17.7	62.5	6.4
4–7	23.7	21.3	23.5	5.8
8–11	12.8	13.8	11.4	7.1
≥ 12	3.5	17.2	2.6	4.3
Wealth				
1st quintile (poorest)	19.5	24.5^c^	20.0	7.2^b^
2nd quintile	19.9	24.6	20.7	8.1
3rd quintile	20.6	20.8	20.2	7.2
4th quintile	18.9	7.4	19.0	4.2
5th quintile	21.2	6.9	20.2	2.3
Ethnicity				
White	53.9	14.7^b^	50.4	5.2^a^
Nonwhite	46.1	21.9	49.6	7.3
Living arrangements				
No marital relationship/doesn’t live alone	44.9	8.9^c^	44.8	4.1^c^
No marital relationship/lives alone	15.1	53.2	15.6	14.4
Marital relationship/doesn’t live alone	40.0	15.0	39.6	5.3
Number of BADL				
1–2	36.6	27.5^c^		
3–4	20.3	21.3		
5–6	43.1	8.5		
Number of IADL				
1			26.9	6.6
2			26.8	6.8
3–4			46.3	5.6

BADL: basic activities of daily life; IADL: instrumental activities of daily life

^a^ p < 0.05

^b^ p < 0.01

^c^ p < 0.001

Table 4 shows the final multiple logistic regression model for the factors related to unmet need. Regarding BADL, non-white individuals and those without a marital relationship and living alone had significantly more chances of having an unmet need (OR = 1.93 and OR = 10.77, respectively). Older individuals with ≥ 80 years had 76% lower chance of having an unmet need when compared with the ones aged 60–79 years old. Being in the 4th and 5th wealth quintiles reduced the chances of having an unmet need. Lower chances of an unmet were also observed among individuals with five or six BADL. Both the factors and direction of association observed regarding BADL were found in relation to IADL, except by the number of IADL, which was not related to unmet need.

**Table 4:** Final regression model for unmet need related to basic (BADL) and instrumental (IADL) activities of daily life.

Variable	Unmet need related to BADL	Unmet need related to IADL

	OR (95%CI)	OR (95%CI)
Sex		
Female	1.40 (0.97–2.02)	0.60 (0.44–0.81)^b^
Age (reference: 60–69 years)		
70–79	0.66 (0.43–1.02)	0.63 (0.44–0.91)^a^
≥ 80	0.24 (0.13–0.42)^c^	0.33 (0.23–0.48)^c^
Ethnicity (reference: white)		
Nonwhite	1.93 (1.28–2.92)^b^	1.29 (0.94–1.77)
Living arrangements (reference; no marital relationship/doesn’t live alone)		
No marital relationship/lives alone	10.77 (6.86–16.91)^c^	3.78 (2.49–5.76)^c^
Marital relationship/doesn’t live alone	1.23 (0.75–1.98)	0.96 (0.63–1.47)
Wealth (reference: 1st quintile)		
2nd quintile	0.84 (0.49-1.44)	1.22 (0.77–1.92)
3rd quintile	1.21 (0.66-2.23)	1.07 (0.69–1.66)
4th quintile	0.47 (0.27-0.81)^b^	1.20 (0.72–2.02)
5th quintile	0.31 (0.15-0.67)^b^	0.39 (0.22–0.68)^b^
Number of BADL (reference: 1–2)		
3–4	0.83 (0.52–1.33)	
5–6	0.28 (0.17–0.45)^c^	

^a^ p < 0.05

^b^ p < 0.01

^c^ p < 0.001

## DISCUSSION

The present study demonstrated that about one-fifth of individuals in need of assistance for BADL had an unmet need, which was higher than the proportion of unmet need for IADL. Unmet needs were significantly related to the individuals’ living arrangements and socioeconomic factors. Unpaid care provided by family members was the most frequent type of caregiver.

Differences in the proportion of unmet need between studies must to be evaluated with caution, as both groups of activities of daily life and unmet need are measured using different definitions. A comparison with Brazilian national estimates is not possible as there is no previous study reported. The available results, from the largest city in Brazil, found that among men and women the lack of care was higher for each BADL than for IADL^17^. Overall, unmet need for BADL was about a third of that observed among Chinese older adults and similar to the ones observed among older US Americans^5^ ^,^ ^12^ and Canadians women^9^. Studies regarding IADL are more restricted, with unmet need ranging from 18%^5^ to 39%^9^, higher than the one found in Brazil.

A higher prevalence of unmet need in BADL than in IADL have been reported^5^ ^,^ ^17^ and is an important issue that needs to be addressed. Having BADL disabilities is associated with a higher probability of receiving assistance and creating a need for a caregiver ^24^. Different from disabilities in IADL, which involves task related to life in the community and are more easily supported by technologies, BADL disabilities are related to activities of self-care and survival and are dependent of direct, hands-on help^25^ ^,^ ^26^, mostly provided by family members^2^ ^,^ ^10^ ^,^ ^24^. Although unpaid care by a family member was the most frequent type of care received by disabled older individuals, the higher proportion of unmet need for BADL may already be pointing out to the changes in family structure and gender relationship in Brazil, which may directly impact the availability of family members to take care of their relatives^2^.

Regarding the correlates of unmet need, the findings corroborate the literature and demonstrated significant association with living arrangements^12^ ^,^ ^14^ ^,^ ^24^ ^,^ ^27^ and socioeconomic and demographic factors ^9,13–15^. Individuals who live alone and don’t have a marital relationship have higher chances of having an unmet need because they lack the strongest supportive tie, the spouse, and are less likely to have children (the next strongest tie)^27^.

Like previous findings^14^ ^,^ ^24^, individuals at higher age groups had lower odds of having an unmet need. These results may rely on the fact that those older adults are the ones with a higher number of disabilities, which, as observed, are also the ones less likely to have unmet need. Previous findings showed no association between BADL disability and the situation of no care^24^ ^,^ ^27^, as people with higher levels of disability cannot remain in the community without help^24^. Although caring for the ones with more need is important, younger individuals with lower levels of disability are the ones most affected by the unmet need as they are living longer with impairment and are at higher risk to become dependent. Accordingly, these older people would benefit from receiving adequate care and interventions to improve their capacity and avoid care dependence^2^, which increases years of living in the community, postponing or averting institutionalization^2^ ^8^.

Socioeconomic inequalities have been observed in different studies^12^ ^,^ ^14^ ^,^ ^24^. In the present study, the probability of unmet need among individuals in the 5th wealth quintile was about half of the ones in the first quintiles. The literature shows that long-term care is unaffordable for most people and the expenditure with services and support represent more than 60% of disposable income among individuals at the bottom 80% of the income distribution^29^. According to the literature, this form of care has an economic cost, which is related to a reduction in labor market participation of relatives and friends^10^. Better economic conditions not only contribute to better health outcomes but also to the availability of paid and family caregivers, with the latter being most likely to afford the burden of leaving the labor market. Thus, long-term care is among the circumstances that need to be adjusted to make changes in socioeconomic and demographic inequalities^30^.

The strengths of this study include the use of a national representative sample from the largest country in Latin America and the assessment of both BADL and IADL providing a more reliable estimate of unmet need, which may be used for planning and supporting long-term care policies. Among the limitations, a lack of causal relationship is expected in cross-sectional studies. Another limitation relies on the fact that unmet need was measured based on self-reported lack of assistance for activities of daily living. Although this assessment is widely used, it does not distinguish the availability of care from its adequacy, thereby leading to some underestimation of unmet need.

This study of a representative sample of Brazilian older individuals highlights that the issue of unmet need should be tackled as a priority among public health policies, considering the large number of individuals and families affected and the existence of socioeconomic inequities. Brazil has high-income inequalities, and although differences between rich and poor have decreased in recent years, inequities in the use of health services have remained significant and may increase the burden of disabilities for this population. Moreover, as care relies mostly on family members, the balance between government and family care provision may not be postponed as families’ patterns and arrangements are changing and this kind of care may not be available for the next generation. Furthermore, long-term care policies may also benefit from future studies directed towards investigating the training, expertise, status, and remuneration of caregivers as a way to improve the quality of help delivered.
